# Estimated prevalence and viral transmissibility in subjects with asymptomatic SARS-CoV-2 infections in Wuhan, China

**DOI:** 10.1093/pcmedi/pbaa032

**Published:** 2020-09-04

**Authors:** Kang Zhang, Weiwei Tong, Xinghuan Wang, Johnson Yiu-Nam Lau

**Affiliations:** Center for BioMedicine and Innovations, Faculty of Medicine, Macau University of Science and Technology, Macao, China; Center for BioMedicine and Innovations, Faculty of Medicine, Macau University of Science and Technology, Macao, China; Zhongnan Hospital of Wuhan University, Wuhan, China; Department of Applied Biology and Chemical Technologies, Hong Kong Polytechnic University, Hung Hom, Hong Kong SAR, China

**Keywords:** asymptomatic carriers, infection, COVID-19

## Abstract

The role of subjects with asymptomatic SARS-CoV-2 infection in the current pandemic is not well-defined. Based on two different approaches to estimate the culminative attack rate (seroprevalence of antibodies against SARS-CoV-2, and a four compartment mathematical model) and the reported number of COVID-19 patients, the ratio of asymptomatic versus symptomatic SARS-CoV-2 infection was estimated to be 7 (95% CI: 2.8–12.4) in Wuhan, Hubei, China, the first epicenter of this pandemic, which has settled with no new cases. Together with detailed recording of the contact sources in a cohort of patients, and applying the estimations to an established mathematical model, the viral transmissibility of the subjects with asymptomatic SARS-CoV-2 infection is around 10% of the symptomatic patients (95% CI: 7.6%–12.3%). Public policies measures/policies should address this important pool of infectious source in our combat of this viral pandemic.

## Main Text:

The role of subjects with asymptomatic SARS-CoV-2 infection in the current pandemic is not yet clear. The ratio of asymptomatic versus symptomatic subjects with SARS-CoV-2 infection was initially estimated to be from 0.22-1.27 times based on viral nucleic acid RT-PCR assay early in the pandemic (reported between February 10 to April 24, 2020, Figure 1). Subsequent studies based on serology for antibodies to SARS-CoV-2, an indicator of immune response related to a past exposure (and reported from April 23 to June 3, 2020) gave a much higher estimation of 8.46-10.24 times (Figure 1). The timing of the study, the study population and the performance of assays could all impact the assessment of

ORIGINAL UNEDITED MANUSCRIPT

the prevalence and as a result, the impact of asymptomatic SARS-CoV-2 infection in its contribution to this pandemic.

Wuhan City in Hubei Province, China is the first epicenter of this pandemic. The epidemic in this city has settled with no new local cases reported for more than two months and therefore, is a good place to look back to determine prevalence and the role of asymptomatic SARS-CoV-2 infection in this pandemic. Timely understanding of the role of asymptomatic SARS-CoV-2 infection is critical as this scientific information may be critical for communities that are still in the midst of this pandemic to fine tune their public health measures/policies. Therefore, we attempted to estimate the prevalence of asymptomatic SARS-CoV-2 infection based on two different approached, the viral transmissibility, the contribution of this pool of infected subjects to the pandemic, and the implications of these information on public healthcare measures/policies.

First, with reference to prevalence, we employed two different approaches to estimate the number of subjects with asymptomatic SARS-CoV-2 infection in Wuhan. We have previously shown a cumulative prevalence of COVID-19 of 3.2%~3.8% in Wuhan estimated based on a survey of serum IgM and IgG levels collected between 9 March, 2020 to April 10, 2020) in 17,368 residents in the city[1]. Based on another survey of 16,101 community subjects who underwent health check-up between March 15 to May 7, 2020) in another hospital in Wuhan, a total of 709 (4.4%) subjects were found to be seropositive for either IgG (n=539), IgM (n=64), or both IgG/IgM (n=106) against SARS-CoV-2. Therefore, the seroprevalance for antibodies SARS-CoV-2 antibodies, or exposure to this virus, is confirmed to be around 4% in Wuhan. Using a different approach, we employed our previously published mathematical model based on a four-compartment modified SQIR (susceptible-quarantined-infected-removed) approach, with the infected compartment subdivided into different status to describe latent (infected but not contagious), asymptomatic (not showing symptom or never showing symptom) and symptomatic individuals[2] (Figure 2). Based on the Wuhan screening data and our model (Figures 2 & 3), we estimate that during the entire course of the outbreak in Wuhan, there were a total of 398,346 infected patients (95% CI: 196,002 – 684,777), or 3.59% (95% CI: 1.77% – 6.17%) of the city population. Both seroprevalence and mathematical modelling approaches provided consistent 3.2 to 4.4% prevalence of SARS-CoV-2 infection, or around 400,000 subjects in total in Wuhan with a population of 11 million. As there were 51,081 symptomatic subjects as reported by Chinese CDC in Wuhan, the balance of 349,000 subjects will be those with asymptomatic SARS-CoV-2 infection. Therefore, the ratio of subjects with asymptomatic SARS-CoV-2 versus symptomatic COVID-19 patients was around 7 (with our mathematical model having 95% CI: 2.8 – 12.4).

Second, we evaluated in detail the contact sources of COVID-19 vs non-COVID-19 patients in Wuhan from Zhongnan Hospital of Wuhan University. Out of 212 patients with COVID-19 admitted to the hospital after the city lockdown, 43.8% had confirmed exposure history (defined as visited Huanan Seafood Wholesale Market, contacted COVID-19 patients, or from the same household of a COVID-19 patient diagnosed before or after). Therefore, 56.2% of the COVID-19 patients with no exposure history might have acquired their viral

ORIGINAL UNEDITED MANUSCRIPT

infection through contact with subjects who had an asymptomatic SARS-COV-2 infection. In contrast, in 571 Non-COVID-19 patients, only 17.1% had history of similar exposure history.

Third, in a revised mathematical model[2], we assumed that the viral transmissibility of asymptomatic infectious subjects ($\sigma_A$) was discounted at a fixed ratio relative to that of symptomatic infected subjects ($\sigma$), and were both affected by general facemask use policy. Using the estimated number of subjects with asymptomatic SARS-CoV-2 infection and the actual number of infection as additional input (Figure 2), we were able to estimate the viral transmissibility (or transmission efficiency) of the subjects with asymptomatic SARS-CoV-2 infection to be around 10% (95% CI: 7.6% – 12.3%) of the symptomatic patients with COVID-19. This is consistent with the hypothesis that subjects that can mount an earlier immune response can control the viral infection better and therefore with less viral load for viral transmission to others. In contrast, subjects without a good early immune response will allow the viral infection to establish a large viral load, which will render the patients more infectious and with more cellular/tissue damage, consequently providing a much severe response later during the clinical course. If our hypothesis is correct, then the reported greater immune response in COVID-19 patients is more a consequence of an earlier weaker response and therefore, a larger viral load and subsequently a higher immune response and tissue damage[3]. The claim of a weaker immune response in asymptomatic individuals in a recent paper, is therefore, the cart rather than the horse[3].

With an estimated ratio of 7x more asymptomatic infection, together with a transmission factor of 10% viral transmissibility compared to symptomatic cases, suggests that the contribution of subjects with asymptomatic SARS-CoV-2 infection represents a significant source for new infections. This is consistent with the report showing that asymptomatic subjects have a lower viral load when first identified but importantly, the duration of viral shedding are similar to COVID-19 patients[4]. Public measures/policies to limit their infection spread once identified will be critical to contain these infection sources. We have previously estimated the impacts of individual public health interventions separately based on the epidemic curves of multiple countries[2]. Both general facemask use (through lowering $\sigma_A$) and social distancing/lockdown (through lowering $\beta$) are effective in mitigating the spread of SARS-CoV-2 from infectious individuals who are asymptomatic, the "dark mass" in the perpetuation of this pandemic.

In addition, if the ratio of 7 asymptomatic to 1 symptomatic SARS-CoV-2 infection is confirmed and applicable to other countries, as of July 19, 2020 (date of submission of this manuscript), the estimated cumulative number of SARS-CoV-2 infection in the US will be close to 30 million, around 16 million in Brazil, and more around 100 million worldwide.

To corroborate with another survey in Wuhan[5-7], our mathematical model estimated that there were still around 897 infected subjects two week after the last reported case in Wuhan in May 18, 2020. Indeed, a government survey in May 2020 on 9.9 million Wuhan residents showed around 300 RT-PCR positive cases[8]. Interestingly, all 1174 close contacts of these RNA positive cases were found to be negative for SARS-CoV-2. Whether these

ORIGINAL UNEDITED MANUSCRIPT

positive cases were infectious and the infection limited by good public polices and general facemask use, which reduce the viral transmissibility factor (based on our model), or whether these subjects only had viral RNA residues or defective interfering particles, remained to be studied.

## Author contributions

K.Z., W.W.T., and J.Y-N.L. contributed to the study design, writing of the manuscript. K.Z., W.W.T., X.H.W., and J.Y-N.L. contributed to data analysis and interpretation. All authors reviewed and approved the final version of the manuscript.

## Competing interests

The authors declare no competing interests.

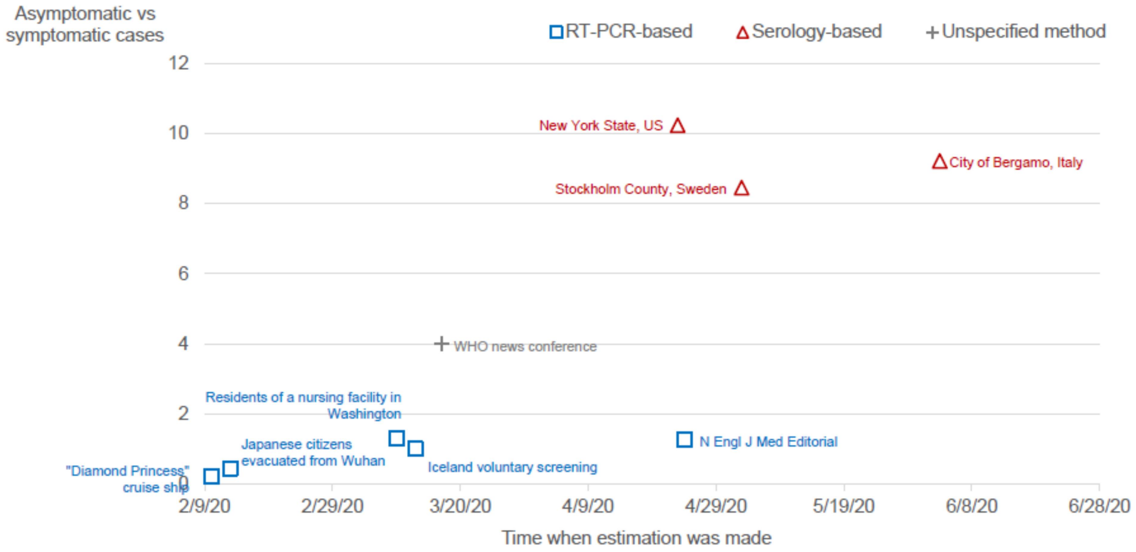

**Figure 1. Various estimations of the ratio between asymptomatic versus symptomatic SARS-CoV-2 infections and the time when these estimations were made.**

Squares represent estimations based on viral RT-PCR test results. Triangles represent estimations based on serology test results in various cities or regions. Cross represents estimation based on unspecified data source.

ORIGINAL UNEDITED MANUSCRIPT

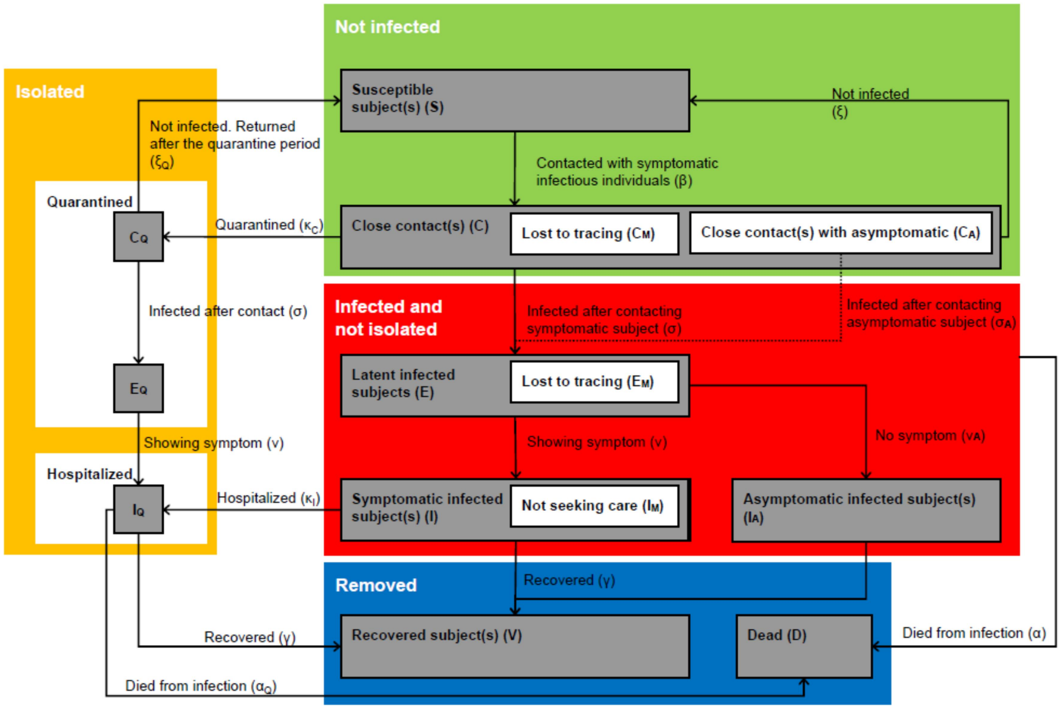

**Figure 2. Revised Flow diagram of the four-compartment mathematical model.**
The four-compartment model of disease transmission incorporates the viral transmissibility and the impact of quarantine and social distancing (revised from our previous model cited in reference 2). The population is divided into the following states: susceptible subject(s) (S), had close contact(s) (C), those that were exposed to the infectious subjects/pathogen but not necessarily infected), latent infected subject(s) (E, infected but not infectious), symptomatic infectious subject(s) (I), asymptomatic infectious subject(s) ($I_A$), recovered (V) and dead (D). $C_M$ is the portion of the contact cases that are missed by contact tracing. $C_A$ is the portion of the cases contacted with asymptomatic infectious subjects (thus remain unknown). Both $C_M$ and $C_A$ will not be quarantined. Individuals in states C and $C_A$ will progress to latent group E. When latent subjects become infectious, the symptomatic subjects are moved to the infectious status I, and asymptomatic to $I_A$. $C_Q$, $E_Q$ and $I_Q$ denote subjects in the quarantine facilities or isolation wards who are quarantined but not necessarily infectious, latent infected and infectious, respectively. Since all subjects under quarantine were regularly tested for SARS-CoV-2, all infected ones were hospitalized regardless of being symptomatic or asymptomatic. It was assumed that when the infected subjects have recovered, they will acquire immunity that does not wane during the timeframe of the analysis (i.e. of this season).

ORIGINAL UNEDITED MANUSCRIPT

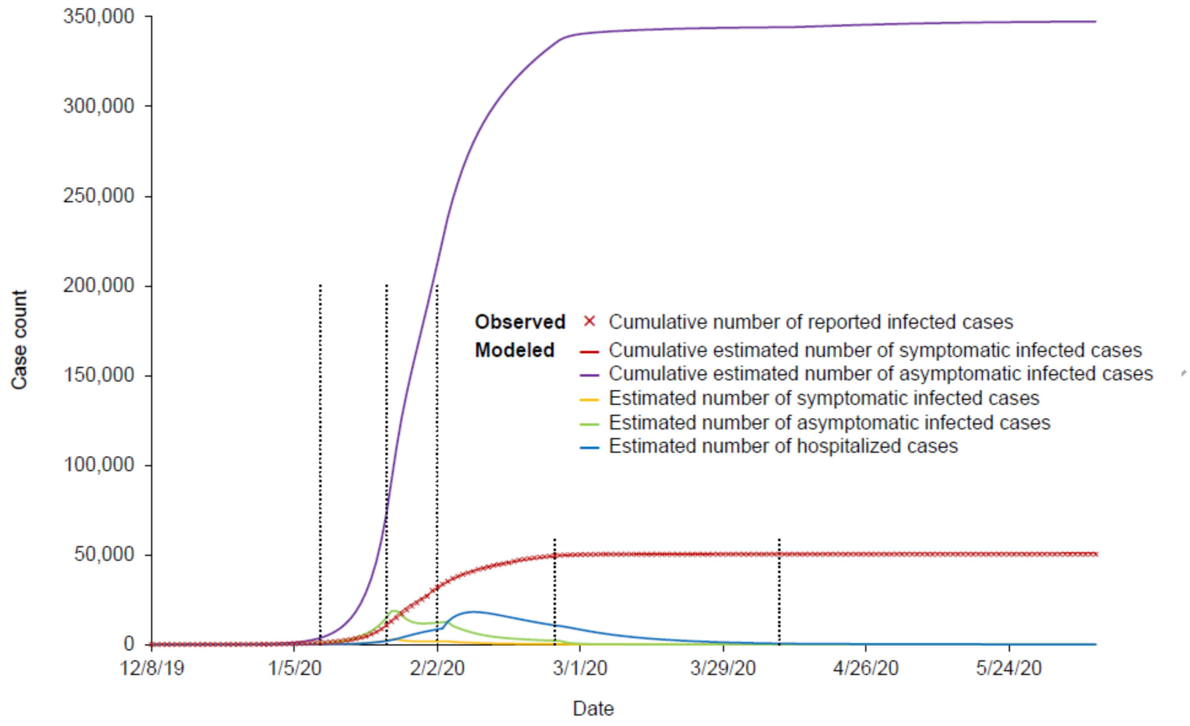

**Figure 3. Observed and estimated case epidemic trajectories in Wuhan, Hubei, China.** A cross symbol represents the cumulative numbers of cases observed. Curves represent the model fitted to the observed data using Maximum Likelihood Estimations (red curve represents the cumulative model-estimated numbers of symptomatic cases, purple curve represents the cumulative model-estimated numbers of asymptomatic cases). Four distinct periods were defined: (a) prior to January 23, 2020 before major public health interventions; (b) between January 23 and January 31, when there was travel ban and cancellation of social gatherings [which would lower per capita contact rates (β)] and compulsory facemask use [which would lower the infection rate upon contact (σ)]; (c) between January 31 and February 17, when quarantine was in place; and (d) between February 17 and July 9, 2020.

ORIGINAL UNEDITED MA
